# In Response

**DOI:** 10.4269/ajtmh.2010.09-0581b

**Published:** 2010-02-05

Dear Sir:

Bryce and Steketee's interest in integrated continuous surveys and quality management^1^ (I-Q) is appreciated, especially because they emphasize its dual potential to strengthen programs and improve monitoring and evaluation systems. They raise several important positive points regarding the flexibility of continuous surveys, the use of time-series analyses to evaluate real-world programs in which public health activities cannot be neatly grouped into intervention and control areas, and the ability of I-Q to address the gap between data, quality, and action at the national and local level. They also raise several important concerns, such as the challenge of creating and maintaining the continuous survey teams, the requirement of a critical mass of supporters and long-term commitment of government leaders and donors, and the risk involved in undertaking such a complex and ambitious strategy.

Although all these concerns are relevant, perhaps the most important is risk. Worries about failure or making a situation worse are natural and healthy; however, they can prevent or delay discoveries of genuinely successful innovations. As Bryce and Stetekee suggest, it is understandable that I-Q could be perceived as being risky because it involves changing parts of the health system to fully implement quality management principles and altering the well-accepted paradigm of periodic cross-sectional household surveys. When considering whether to risk an intervention with potentially far-reaching consequences, one might ask: are we content with the status quo? In countries with increased resources, are interventions being scaled-up as quickly and equitably as desired? Are existing monitoring and evaluation systems efficiently providing the needed information on coverage and impact to programs and donors? On this last question, consider the example of one country, in which five nationwide, cross-sectional household surveys were conducted in a 12-month period.^2^ Altogether these surveys collected data from nearly 35,000 households (about five times larger than a typical Demographic and Health Survey^3^); and although each survey lasted 1–5 months, data collection occurred in every month of the 12-month period—essentially continuous data collection. Other similar examples exist.

Of course, the middle path that balances the risks of failure and missing a successful innovation involves starting with a sub-national pilot project, retaining any existing plans for conventional cross-sectional surveys, and evaluating the pilot experience carefully before deciding whether to scale-up. In such a pilot study of I-Q, four recommendations should be considered. First, test the entire concept: do not only implement continuous surveys without the quality management component. While continuous surveys might have some advantages over periodic cross-sectional surveys, the flow of so much data without improved structures to use the results is potentially wasteful, and such an approach does not truly test the I-Q concept. Similarly, health facility surveys should be included, as they can provide valuable information on the quality of care and availability of commodities over time. For example, health facility surveys on malaria in low-income African countries revealed that despite reasonably well-designed efforts to implement new policies on testing and treatment, underuse of malaria testing was common, negative test results were often ignored, and many patients needing antimalarial therapy did not receive it.^4^–^10^

The second recommendation is to test the concept in a reasonably large geographic area, perhaps at least 10 districts. A test in only a few districts will make the surveys seem disproportionately expensive (in terms of cost per population size) and overly precise (surveys could produce sub-district results, which would not be feasible if I-Q was scaled-up nationally). Furthermore, the challenge of implementing quality management principles might not be fully experienced in small areas because distances are short, populations more likely to be similar, and everything is logistically easier. The third recommendation is to let a pilot study continue long enough to allow for a full evaluation, perhaps at least 5 years. As Bryce and Steketee rightly point out, it will take time to establish the continuous survey teams and quality management structures. Finally, adequate funding is essential to ensure a sufficient number of survey teams, robust quality management structures, and resources for quality improvement teams to implement changes. It would be better to test a fully funded I-Q project that was successful and then think of ways to reduce costs than to have an underfunded pilot that does not realize its potential.

Alexander K. Rowe

Malaria Branch

Division of Parasitic Diseases

Centers for Disease Control and Prevention

Atlanta, GA 30341-3724

E-mail: axr9@cdc.gov
